# Effects of carnosic acid on renal injury-related parameters and iNOS immunoreactivity in an experimental model of contrast-associated acute kidney injury

**DOI:** 10.1080/0886022X.2026.2719144

**Published:** 2026-08-26

**Authors:** Nejmi Küçükyağcı, Ömer Salt, Mustafa Burak Sayhan, Nurettin Aydoğdu, Ebru Taştekin, Eray Çeliktürk, Esin Seçgin Sayhan

**Affiliations:** ^a^ Emergency Department, Artvin State Hospital, Artvin, Turkey; ^b^ Emergency Department, Kayseri Training and Research Hospital, Edirne, Turkey; ^c^ Department of Emergency Medicine, Trakya University Medicine Faculty, Edirne, Turkey; ^d^ Department of Physiology Inonu University Faculty of Medicine, Malatya, Turkey; ^e^ Department of Medical Pathology, Trakya University Medicine Faculty, Edirne, Turkey; ^f^ Department of Public Health, Trakya University Medicine Faculty, Edirne, Turkey

**Keywords:** Contrast-associated acute kidney injury, carnosic acid, inducible nitric oxide synthase, oxidative stress, renal tubular injury

## Abstract

Contrast-associated acute kidney injury (CA-AKI) remains a clinically relevant complication of iodinated contrast exposure. Carnosic acid possesses antioxidant and anti-inflammatory properties, but its effects on renal biochemical injury, tubular damage, and inducible nitric oxide synthase (iNOS) immunoreactivity in CA-AKI remain unclear. This study evaluated carnosic acid in an experimental rat model of CA-AKI. Twenty-four adult male rats were randomly assigned to control, CA-AKI, or CA-AKI plus carnosic acid groups (*n* = 8 each). CA-AKI was induced by 24-h restriction, furosemide, and intravenous iohexol. After contrast exposure, carnosic acid was administered therapeutically at 30 mg/kg once daily for 5 days through an orogastric catheter. Serum urea, creatinine, sodium, potassium, and creatine kinase levels were measured. Tubular injury was scored semi-quantitatively, and iNOS immunoreactivity was assessed using the H-score. Groups were compared using the Kruskal–Wallis test with Dunn–Bonferroni post hoc analysis. Compared with controls, the CA-AKI group had significantly higher urea, creatinine, potassium, creatine kinase, tubular injury scores, and iNOS H-scores and lower sodium levels. Compared with untreated CA-AKI, carnosic acid treatment significantly reduced urea and creatine kinase levels and increased sodium levels. Creatinine concentrations, tubular injury scores, and iNOS H-scores were numerically lower after treatment, but pairwise differences were not statistically significant. Thus, carnosic acid improved selected biochemical parameters, while its apparent histopathological and iNOS-related effects remained inconclusive. These findings support a possible but limited protective effect in this experimental setting. Further studies incorporating molecular validation and dose and timing protocols are required to clarify its renoprotective potential and mechanisms.

## GRAPHICAL ABSTRACT

**Figure:**
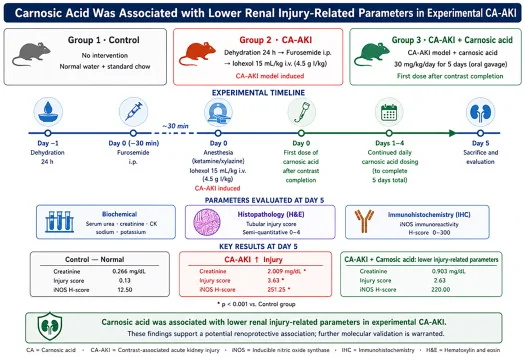

## Introduction

Iodinated contrast agents are widely used in diagnostic and interventional procedures and constitute an essential component of modern clinical practice [1–3]. Nevertheless, contrast-associated acute kidney injury (CA-AKI) remains a clinically relevant nephrological concern [1,2]. CA-AKI is commonly defined as an increase in serum creatinine of ≥0.3 mg/dL or ≥50% from baseline within 48–72 h after contrast exposure [4,5]. Renal dysfunction occurring after contrast administration is associated with adverse clinical outcomes, particularly in patients with reduced renal reserve, hemodynamic instability, or significant comorbidities [3,4,6].

Current literature emphasizes that not every episode of renal dysfunction observed after contrast exposure can be directly attributed to contrast nephrotoxicity. Therefore, it has been recommended to distinguish between ‘CA-AKI’, which denotes a temporal association with contrast exposure, and ‘contrast-induced acute kidney injury’, which implies a causal role of contrast media [4,5]. In the present study, the term CA-AKI was used to maintain consistency with contemporary clinical terminology.

The pathophysiology of CA-AKI is multifactorial. Renal medullary hypoxia, intrarenal vasoconstriction, tubular cytotoxicity, oxidative stress, and inflammatory activation act together in this process [7–9]. Among these mechanisms, oxidative stress and inflammation have an important role in the development of tubular epithelial injury [8,9]. In this context, inducible nitric oxide synthase (iNOS) is regarded as an inflammatory mediator associated with renal microcirculatory dysfunction and tubular cell injury through increased nitric oxide production and nitrosative stress [10]. Therefore, evaluation of the iNOS-related inflammatory response is biologically relevant for understanding tissue-level injury in CA-AKI.

In current clinical practice, intravenous hydration is considered the main preventive strategy for reducing the risk of CA-AKI [1,5,7]. However, alternative pharmacological agents with efficacy supported by strong evidence remain limited. Considering the role of oxidative and inflammatory mechanisms in the pathogenesis of CA-AKI, experimental investigation of agents targeting these pathways is scientifically relevant.

Carnosic acid is a phenolic diterpene compound derived from *Rosmarinus officinalis* and is known for its antioxidant and anti-inflammatory properties. Previous experimental studies in renal injury models, including cisplatin-induced nephrotoxicity, cadmium-induced nephrotoxicity, and lipopolysaccharide-induced acute kidney injury, have reported favorable effects of carnosic acid on renal dysfunction and injury-related pathways such as oxidative stress, inflammatory activation, apoptosis, and tubular injury [11–13]. However, data evaluating the effects of carnosic acid on renal function, histopathological tubular injury, and iNOS immunoreactivity together in the context of CA-AKI remain limited. This gap in the literature formed the main rationale for the present study. This study aimed to evaluate the effects of carnosic acid administration on renal function parameters, histopathological tubular injury, and iNOS immunoreactivity in an experimental model of CA-AKI.

## Methods

### Experimental design and animals

This experimental study was approved by the Local Ethics Committee for Animal Experiments of Trakya University (Approval No: 2018.12.01). All experimental procedures were conducted in accordance with national and international ethical principles for animal experimentation. The study was designed and reported with consideration of the ARRIVE 2.0 reporting guidelines.

Adult male rats weighing 250–300 g were used in the study. Animals were housed under standard laboratory conditions at a room temperature of 21 ± 4 °C and a 12-h light/12-h dark cycle. Throughout the experimental period, animals had *ad libitum* access to standard rat chow and water, except during the fluid restriction period.

Rats were allocated into three groups using simple randomization, with eight animals in each group. Group 1 was designated as the control group, Group 2 as the CA-AKI group, and Group 3 as the CA-AKI + carnosic acid group. The CA-AKI model was not applied to the control group. In the CA-AKI group, an experimental model of CA-AKI was established. In the CA-AKI + carnosic acid group, carnosic acid was administered after establishment of the same model. No animals were excluded from the analysis.

### Establishment of the experimental CA-AKI model

In this study, to maintain terminological consistency, the experimental model was referred to as a CA-AKI model in accordance with the ACR/NKF consensus statement [5]. The model was established under controlled experimental conditions using dehydration, furosemide administration, and iohexol administration, as described in detail below.

In rats assigned to the CA-AKI and CA-AKI + carnosic acid groups, dehydration was induced by restricting oral fluid intake for 24 h before contrast agent administration. After this period, furosemide was administered intraperitoneally at a dose of 10 mg/kg to reduce renal perfusion. Thirty minutes after furosemide administration, anesthesia was induced with intramuscular ketamine 90 mg/kg and xylazine 10 mg/kg [14].

After anesthesia, iohexol, a nonionic low-osmolar iodinated contrast agent (Omnipaque 300 mg I/mL; GE Healthcare Ireland Limited, Cork, Ireland), was administered intravenously *via* the tail vein. The iohexol dose was set at 15 mL/kg, corresponding to approximately 4.5 g iodine/kg according to the iodine concentration of the preparation used. The administration rate was standardized at 0.5 mL/min. Thus, the experimental CA-AKI model was established. This protocol was applied in accordance with experimental acute kidney injury models induced by iodinated contrast media in rats [15]. After completion of contrast agent administration, fluid restriction was discontinued, and all animals were allowed *ad libitum* access to water and food.

### Carnosic acid administration

Carnosic acid was administered only to animals in the CA-AKI + carnosic acid group. In this study, carnosic acid was planned as a therapeutic intervention initiated after model induction; no prophylactic administration was performed. Carnosic acid (≥91% purity; Sigma–Aldrich, St. Louis, MO, USA; Cat. No. C0609) was dissolved in corn oil and administered *via* orogastric catheter at a dose of 30 mg/kg once daily for 5 days following completion of contrast agent administration on the day of model induction. Control and CA-AKI group animals did not receive carnosic acid or corn oil vehicle. This dose and treatment schedule were selected by adapting previously reported experimental carnosic acid protocols in kidney injury models to the present CA-AKI model [11–13].

### Sample collection and tissue preparation

At the end of the 5-day carnosic acid administration period (day 5 after model induction), all rats were deeply anesthetized with intramuscular ketamine hydrochloride (90 mg/kg) and xylazine hydrochloride (10 mg/kg). While under deep anesthesia, blood samples were collected by cardiac puncture. Blood samples were centrifuged at 3000 rpm for 10 min for biochemical analyses, and serum samples were separated and stored at −80 °C until analysis. Both kidneys were rapidly excised through an anterior abdominal incision and longitudinally divided into two parts. The animals were then euthanized by cervical dislocation while still under deep anesthesia. One portion of the kidney tissue was fixed in 10% neutral buffered formalin and embedded in paraffin for histopathological and immunohistochemical analyses; the remaining portion was stored at −80 °C for potential further analyses. All anesthesia and euthanasia procedures were conducted in accordance with the protocol approved by the Trakya University Local Ethics Committee for Animal Experiments (approval no. 2018.12.01) and applicable institutional animal welfare and veterinary guidelines.

### Histopathological assessment and scoring

Sections of 4–5 µm thickness were obtained from paraffin-embedded renal tissues and stained with hematoxylin and eosin. The sections were evaluated under a light microscope by an experienced pathologist who was blinded to the experimental groups. Renal tubular injury was semi-quantitatively scored by considering epithelial cell degeneration, cytoplasmic vacuolization, brush border loss, tubular dilatation, and the presence of intraluminal debris. The degree of injury was classified from 0 to 4 according to the percentage of affected tubules: 0, normal histological architecture; 1, ≤25% involvement; 2, 26–50% involvement; 3, 51–75% involvement; and 4, 76–100% involvement. For each sample, at least 10 randomly selected microscopic fields were evaluated, and the mean injury score was calculated.

### Immunohistochemical assessment

Immunohistochemical staining was performed on paraffin-embedded sections to evaluate iNOS immunoreactivity. After deparaffinization and rehydration, antigen retrieval was performed using an appropriate buffer solution. Endogenous peroxidase activity was blocked with 3% hydrogen peroxide, and a blocking solution was used to prevent nonspecific binding. Sections were incubated with a primary antibody against iNOS (Bioss, Woburn, MA, USA; catalog no. AH02023741; dilution 1:200) at 37 °C for 90 min. Subsequently, an appropriate secondary antibody and streptavidin–peroxidase system were applied. Immunoreactivity was visualized using 3,3′-diaminobenzidine as the chromogen, and nuclei were counterstained with hematoxylin. All sections were processed under the same protocol conditions.

Immunohistochemical evaluation was performed under a light microscope by the same pathologist who was blinded to the experimental groups. iNOS immunoreactivity was assessed using the H-score method by considering both staining intensity and the percentage of positively stained cells. Staining intensity was classified as 0, negative; 1, weak; 2, moderate; and 3, strong. The percentage of positively stained cells and staining intensity were evaluated together, and an H-score ranging from 0 to 300 was calculated. For each sample, at least 10 randomly selected microscopic fields were examined, and the mean score was recorded.

### Biochemical assessment

Serum urea, creatinine, sodium, potassium, and creatine kinase levels were measured using an automated biochemical analyzer with spectrophotometric methods (AU5800, Beckman Coulter Inc., Brea, CA, USA). All biochemical measurements were performed using the same analyzer and reagents, in accordance with the manufacturer’s instructions. The primary outcome measures of the study were renal tubular injury score and iNOS immunoreactivity score. Secondary outcome measures were defined as serum biochemical parameters.

### Statistical analysis

Statistical analyses were performed using IBM SPSS Statistics version 22.0 (IBM Corp., Armonk, NY, USA). The distributional characteristics of numerical variables were assessed using the Shapiro–Wilk test. Data are presented as mean ± standard deviation and median (25th–75th percentile). Comparisons among the three groups were performed using the Kruskal–Wallis test. For variables showing a significant difference in the Kruskal–Wallis test, pairwise group comparisons were performed using the Dunn–Bonferroni *post hoc* test. Effect sizes for Kruskal–Wallis analyses were reported using epsilon-squared (*ε*^2^). Exact p values were reported whenever possible; results with *p* < 0.001 were reported as such. A *p* value of <0.05 was considered statistically significant. No formal sample size calculation or statistical power analysis was performed before the study. Eight animals per group were planned. This issue is further addressed in the limitations section.

## Results

Three experimental groups were compared in this study: the control group, the CA-AKI group, and the CA-AKI + carnosic acid group. Each group included eight animals. Biochemical parameters, histopathological tubular injury scores, and iNOS immunoreactivity scores obtained on day 5 were evaluated across the groups.

### Biochemical findings

Serum urea levels differed significantly among the groups (Kruskal–Wallis *H* = 15.459, *p* < 0.001, ε^2^ = 0.641). Urea levels were significantly higher in the CA-AKI group than in the control group (115.38 ± 39.91 mg/dL vs. 46.75 ± 3.37 mg/dL; Dunn–Bonferroni-adjusted *p* = 0.003). In the CA-AKI + carnosic acid group, urea levels were significantly lower than those in the CA-AKI group (45.88 ± 4.73 mg/dL vs. 115.38 ± 39.91 mg/dL; adjusted *p* = 0.001). No significant difference in urea levels was observed between the control group and the CA-AKI + carnosic acid group (adjusted *p* = 1.000).

Serum creatinine levels differed significantly among the groups (Kruskal–Wallis *H* = 20.498, *p* < 0.001, ε^2^ = 0.881). Creatinine levels were significantly higher in the CA-AKI group than in the control group (2.009 ± 0.343 mg/dL vs. 0.266 ± 0.035 mg/dL; Dunn–Bonferroni-adjusted *p* < 0.001). In the CA-AKI + carnosic acid group, creatinine levels were numerically lower than those in the CA-AKI group (0.903 ± 0.230 mg/dL vs. 2.009 ± 0.343 mg/dL); however, this difference did not reach statistical significance in the pairwise comparison (adjusted *p* = 0.071). The difference between the control group and the CA-AKI + carnosic acid group was also not statistically significant in the pairwise comparison (adjusted *p* = 0.071).

Serum sodium levels differed significantly among the groups (Kruskal–Wallis *H* = 14.813, *p* = 0.001, ε^2^ = 0.610). Sodium levels were significantly lower in the CA-AKI group than in the control group (129.00 ± 8.23 mmol/L vs. 140.63 ± 2.07 mmol/L; Dunn–Bonferroni-adjusted *p* = 0.001). In the CA-AKI + carnosic acid group, sodium levels were significantly higher than those in the CA-AKI group (139.75 ± 1.83 mmol/L vs. 129.00 ± 8.23 mmol/L; adjusted *p* = 0.015). No significant difference in sodium levels was observed between the control group and the CA-AKI + carnosic acid group (adjusted *p* = 1.000).

Serum potassium levels also differed significantly among the groups (Kruskal–Wallis *H* = 9.564, *p* = 0.008, ε^2^ = 0.360). Potassium levels were significantly higher in the CA-AKI group than in the control group (5.69 ± 0.74 mmol/L vs. 4.60 ± 0.43 mmol/L; Dunn–Bonferroni-adjusted *p* = 0.008). In the CA-AKI + carnosic acid group, potassium levels were numerically lower than those in the CA-AKI group; however, this difference did not reach statistical significance in the pairwise comparison (4.85 ± 0.31 mmol/L vs. 5.69 ± 0.74 mmol/L; adjusted *p* = 0.109). No significant difference in potassium levels was observed between the control group and the CA-AKI + carnosic acid group (adjusted *p* = 1.000).

Serum creatine kinase levels differed significantly among the groups (Kruskal–Wallis *H* = 18.240, *p* < 0.001, *ε*^2^ = 0.773). CK levels were significantly higher in the CA-AKI group than in the control group (1547.13 ± 269.65 IU/L vs. 773.13 ± 115.88 IU/L; Dunn–Bonferroni-adjusted *p* < 0.001). In the CA-AKI + carnosic acid group, CK levels were significantly lower than those in the CA-AKI group (965.88 ± 138.04 IU/L vs. 1547.13 ± 269.65 IU/L; adjusted *p* = 0.033). No significant difference in CK levels was observed between the control group and the CA-AKI + carnosic acid group (adjusted *p* = 0.269).

**Figure 1.**
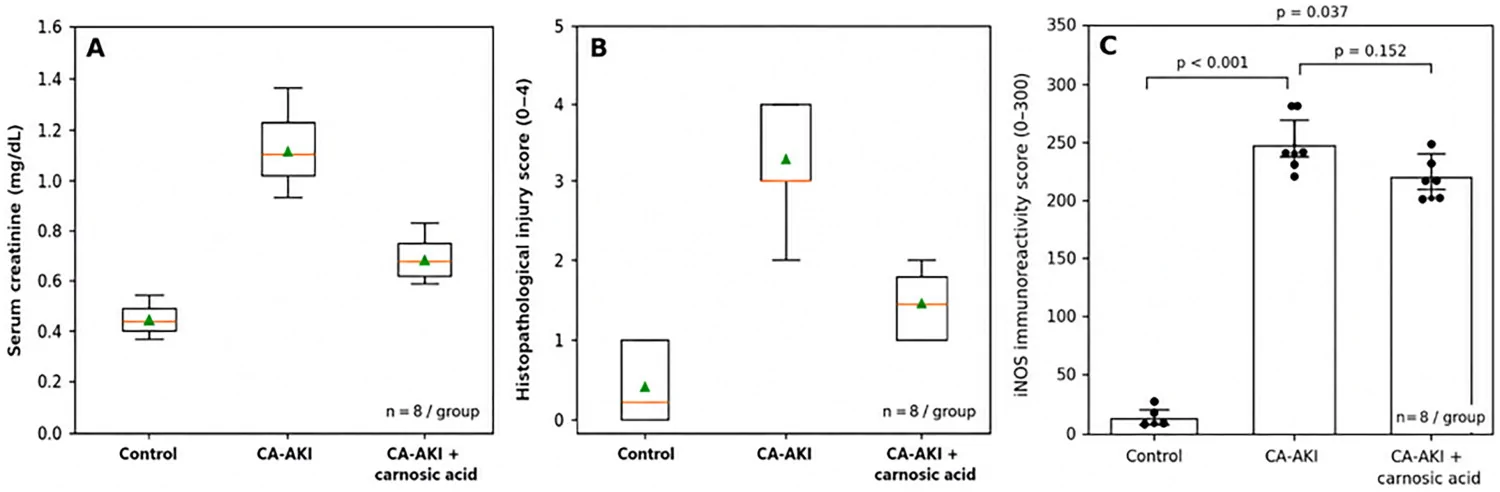
Comparison of key biochemical, histopathological, and immunohistochemical outcomes among experimental groups. (A) Serum creatinine levels (mg/dL) shown as box-and-whisker plots. (B) Semi-quantitative renal tubular injury scores (0–4) shown as box-and-whisker plots. (C) Renal iNOS immunoreactivity H-scores (0–300) shown as bar plots with individual data points and Dunn–Bonferroni-adjusted pairwise comparison *p* values. In box-and-whisker plots, the horizontal line within each box represents the median, the box represents the interquartile range, whiskers indicate the data range, and triangles indicate the mean. In panel C, bars represent mean ± SD. Group comparisons were performed using the Kruskal–Wallis test, followed by Dunn–Bonferroni *post hoc* tests where appropriate. Although iNOS H-scores differed significantly among the three groups overall, the difference between the CA-AKI and CA-AKI + carnosic acid groups did not reach statistical significance. CA-AKI, contrast-associated acute kidney injury; iNOS, inducible nitric oxide synthase; SD, standard deviation.

Group values, Kruskal–Wallis *p* values, effect sizes, and Dunn–Bonferroni-adjusted pairwise comparison results for biochemical parameters are presented in Table 1. The distribution of serum creatinine levels across the groups is shown in Figure 1(A).

**Table 1.** Biochemical parameters according to experimental groups.

Parameter	Control group (*n* = 8)	CA-AKI group (*n* = 8)	CA-AKI + carnosic acid group (*n* = 8)	Kruskal–Wallis H	*p* value	*ε*²	Control vs. CA-AKI	Control vs. CA-AKI + carnosic acid	CA-AKI vs. CA-AKI + carnosic acid
Urea (mg/dL)	46.75 ± 3.40; 45.5 (44.25–49.50)	115.38 ± 39.91; 101.5 (85.50–159.00)	45.88 ± 4.73; 47.0 (41.25–50.00)	15.459	<0.001	0.641	0.003	1.000	0.001
Creatinine (mg/dL)	0.266 ± 0.035; 0.255 (0.24–0.30)	2.009 ± 0.343; 1.905 (1.71–2.27)	0.903 ± 0.230; 0.925 (0.74–1.09)	20.498	<0.001	0.881	<0.001	0.071	0.071
Sodium (mmol/L)	140.63 ± 2.07; 141.0 (140.25–141.75)	129.00 ± 8.23; 130.0 (124.50–135.50)	139.75 ± 1.83; 140.5 (138.50–141.00)	14.813	0.001	0.610	0.001	1.000	0.015
Potassium (mmol/L)	4.60 ± 0.43; 4.65 (4.15–5.00)	5.69 ± 0.74; 5.55 (4.95–6.45)	4.85 ± 0.31; 4.90 (4.63–5.10)	9.564	0.008	0.360	0.008	1.000	0.109
Creatine kinase (IU/L)	773.13 ± 115.88; 764.0 (656.50–871.25)	1547.13 ± 269.65; 1488.5 (1356.75–1750.25)	965.88 ± 138.04; 972.0 (876.50–1024.75)	18.240	<0.001	0.773	<0.001	0.269	0.033

Data are presented as mean ± SD; median (25th–75th percentile). Overall group comparisons were performed using the Kruskal–Wallis test. Pairwise comparisons were performed using Dunn–Bonferroni *post hoc* tests, and adjusted p values are shown. *ε*^2^ indicates the effect size for the Kruskal–Wallis test. CA-AKI, contrast-associated acute kidney injury; SD, standard deviation.

### Histopathological findings

Renal tubular injury scores differed significantly among the groups (Kruskal–Wallis *H* = 19.771, *p* < 0.001, *ε*^2^ = 0.846). The tubular injury score was low in the control group (0.13 ± 0.35), markedly increased in the CA-AKI group (3.63 ± 0.52), and numerically lower in the CA-AKI + carnosic acid group than in the CA-AKI group (2.63 ± 0.52). In Dunn–Bonferroni pairwise comparisons, significant differences were observed between the control and CA-AKI groups (adjusted *p* < 0.001) and between the control and CA-AKI + carnosic acid groups (adjusted *p* = 0.026). The difference between the CA-AKI and CA-AKI + carnosic acid groups did not reach statistical significance (adjusted *p* = 0.217).

Normal tubular and glomerular morphology was observed in the control group. In the CA-AKI group, tubular dilation, epithelial degeneration, and structural disorganization were prominent. In the CA-AKI + carnosic acid group, these histopathological alterations appeared less severe than those observed in the CA-AKI group.

**Figure 2.**
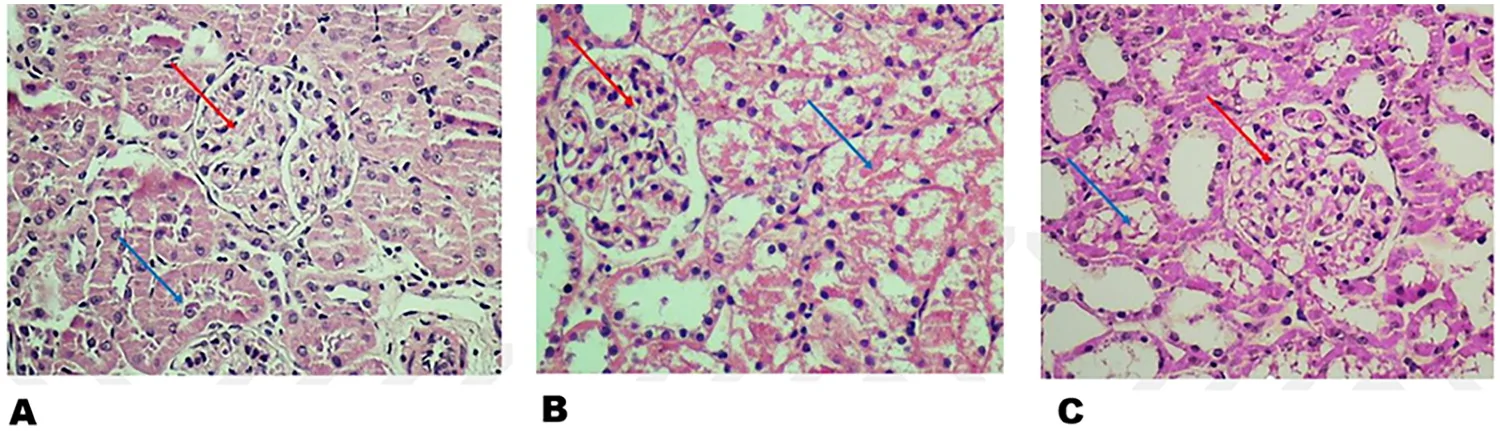
Representative renal histopathological sections from the experimental groups. (A) Control group showing preserved glomerular and tubular architecture. (B) CA-AKI group showing marked tubular dilation, epithelial degeneration, brush border loss, and structural disorganization. (C) CA-AKI + carnosic acid group showing less severe tubular morphological alterations compared with the CA-AKI group. Hematoxylin and eosin staining. Arrows indicate tubular dilation, and asterisks indicate epithelial degeneration and brush border loss. CA-AKI, contrast-associated acute kidney injury.

Histopathological injury scores, the Kruskal–Wallis p value, and the effect size are presented in Table 2. The distribution of renal tubular injury scores across the groups is shown in Figure 1(B). Representative histopathological sections are shown in Figure 2.

**Table 2.** Histopathological tubular injury scores and statistical comparisons according to experimental groups.

Group	Score 0, *n* (%)	Score 1, *n* (%)	Score 2, *n* (%)	Score 3, *n* (%)	Score 4, *n* (%)	Mean ± SD	Median (25th–75th percentile)
Control group (*n* = 8)	7 (87.5)	1 (12.5)	0 (0.0)	0 (0.0)	0 (0.0)	0.13 ± 0.35	0.0 (0.0–0.0)
CA-AKI group (*n* = 8)	0 (0.0)	0 (0.0)	0 (0.0)	3 (37.5)	5 (62.5)	3.63 ± 0.52	4.0 (3.0–4.0)
CA-AKI + carnosic acid group (*n* = 8)	0 (0.0)	0 (0.0)	3 (37.5)	5 (62.5)	0 (0.0)	2.63 ± 0.52	3.0 (2.0–3.0)
*Statistical comparison*			*Test/statistic*			*p value*	*Effect size*
Overall group comparison			Kruskal–Wallis *H* = 19.771			<0.001	*ε*² = 0.846
Control vs CA-AKI			Dunn–Bonferroni post hoc test			<0.001	—
Control vs CA-AKI + carnosic acid			Dunn–Bonferroni post hoc test			0.026	—
CA-AKI vs CA-AKI + carnosic acid			Dunn–Bonferroni post hoc test			0.217	—

Data are presented as count (%), mean ± SD, and median (25th–75th percentile). Histopathological tubular injury was scored semi-quantitatively from 0 to 4 according to the percentage of affected tubules: 0, normal histological architecture; 1, ≤25% involvement; 2, 26–50% involvement; 3, 51–75% involvement; and 4, 76–100% involvement. Overall group comparison was performed using the Kruskal–Wallis test. Pairwise comparisons were performed using Dunn–Bonferroni *post hoc* tests, and adjusted *p* values are shown in the table. *ε*^2^ indicates the effect size for the Kruskal–Wallis test. CA-AKI, contrast-associated acute kidney injury; SD, standard deviation.

## Immunohistochemical findings

iNOS immunoreactivity scores differed significantly among the groups (Kruskal–Wallis *H* = 19.932, *p* < 0.001, *ε*^2^ = 0.854). The iNOS score was significantly higher in the CA-AKI group than in the control group (251.25 ± 15.53 vs. 12.50 ± 4.63; Dunn–Bonferroni-adjusted *p* < 0.001). In the CA-AKI + carnosic acid group, the iNOS score was numerically lower than that in the CA-AKI group (220.00 ± 13.09 vs. 251.25 ± 15.53); however, this difference did not reach statistical significance in the pairwise comparison (adjusted *p* = 0.152). The iNOS score was also significantly higher in the CA-AKI + carnosic acid group than in the control group (adjusted *p* = 0.037).

Minimal iNOS immunoreactivity was observed in the control group. In the CA-AKI group, prominent cytoplasmic iNOS staining was observed in tubular epithelial cells. In the CA-AKI + carnosic acid group, iNOS staining intensity appeared lower than that in the CA-AKI group; however, this difference did not reach statistical significance at the score level in the pairwise comparison.

**Figure 3.**
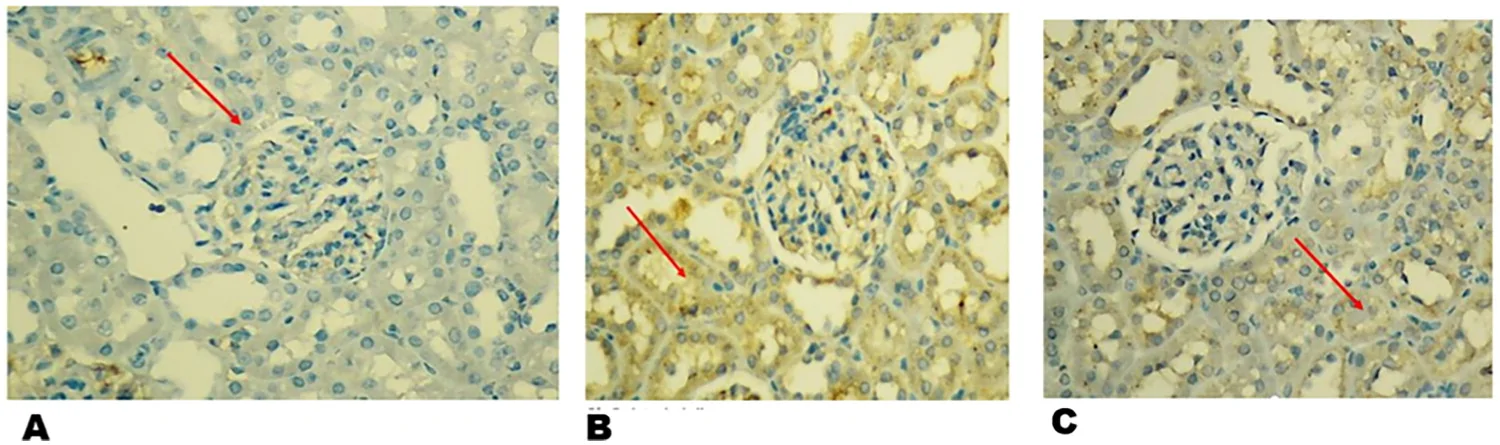
Representative renal iNOS immunohistochemical staining from the experimental groups. (A) Control group showing minimal iNOS immunoreactivity. (B) CA-AKI group showing marked cytoplasmic iNOS staining in tubular epithelial cells. (C) CA-AKI + carnosic acid group showing visually lower iNOS staining intensity compared with the CA-AKI group; however, the quantitative H-score difference between these two groups did not reach statistical significance in pairwise post hoc analysis. Arrows indicate tubular epithelial cells with strong iNOS positivity. CA-AKI, contrast-associated acute kidney injury; iNOS, inducible nitric oxide synthase.

iNOS immunoreactivity scores, the Kruskal–Wallis *p* value, and effect size are presented in Table 3. Dunn–Bonferroni-adjusted pairwise comparison *p* values for iNOS H-scores are also displayed in Figure 1(C) to facilitate visual interpretation. Representative iNOS immunohistochemical staining examples are shown in Figure 3.

**Table 3.** Renal iNOS H-scores according to experimental groups.

Parameter	Control group (*n* = 8)	CA-AKI group (*n* = 8)	CA-AKI + carnosic acid group (*n* = 8)	Kruskal–Wallis H	*p* value	*ε*²
iNOS H-score	12.50 ± 4.63; 10.0 (10.0–17.5)	251.25 ± 15.53; 240.0 (240.0–270.0)	220.00 ± 13.09; 215.0 (210.0–235.0)	19.932	<0.001	0.854

Data are presented as mean ± SD and median (25th–75th percentile). Overall group comparison was performed using the Kruskal–Wallis test. Dunn–Bonferroni-adjusted pairwise comparison *p* values are illustrated in Figure 1(C). *ε*^2^ indicates the effect size for the Kruskal–Wallis test. iNOS immunoreactivity was evaluated using the H-score method (0–300). CA-AKI, contrast-associated acute kidney injury; iNOS, inducible nitric oxide synthase; SD, standard deviation.

## Discussion

In this experimental study, carnosic acid administration after contrast exposure was associated with significant improvement in selected biochemical parameters, specifically urea, sodium, and creatine kinase. Serum creatinine concentrations, histopathological tubular injury scores, and iNOS H-scores were numerically lower in the carnosic acid-treated group than in the untreated CA-AKI group; however, these pairwise differences did not reach statistical significance. Therefore, the histopathological and immunohistochemical findings should be interpreted as non-significant numerical trends rather than definitive treatment effects. Overall, the findings suggest a possible biochemical benefit of carnosic acid in this experimental CA-AKI model, but they do not establish a definite histopathological or iNOS-mediated protective effect.

The apparent discrepancy between significant improvement in selected biochemical parameters and the absence of statistically significant pairwise differences in serum creatinine, tubular injury scores, and iNOS H-scores may be explained by several factors. Biochemical parameters may respond more rapidly to short-term changes in renal perfusion, tubular handling, and systemic injury-related stress, whereas histopathological injury and immunohistochemical signal changes may require a longer observation period to become clearly distinguishable between groups. In addition, the small group size and the semi-quantitative nature of histopathological and iNOS assessments may have limited the ability to detect statistically significant between-group differences despite numerically lower values in the carnosic acid-treated group.

The pathophysiology of CA-AKI is multifactorial and involves renal medullary hypoxia, intrarenal vasoconstriction, osmotic and direct tubular toxicity, oxidative stress, and inflammatory activation [4,7–9, 16–18]. These interconnected mechanisms contribute to tubular epithelial injury and renal microcirculatory dysfunction after iodinated contrast exposure. Within this framework, nitric oxide synthase pathways, including inducible nitric oxide synthase, have been implicated in acute renal injury through altered nitric oxide production, microvascular dysfunction, and nitrosative stress [10]. In the present study, the increased iNOS immunoreactivity observed in the CA-AKI group is consistent with this inflammatory/nitrosative injury framework; however, because iNOS was assessed only by semi-quantitative immunohistochemistry, this finding should be interpreted as supportive tissue-level evidence rather than proof of a causal iNOS-mediated mechanism.

Carnosic acid has been evaluated in previous experimental kidney injury models as an agent with antioxidant and anti-inflammatory properties. In cisplatin-induced nephrotoxicity and cadmium-induced nephrotoxicity, carnosic acid was reported to improve renal injury-related parameters and modulate oxidative stress, lipid peroxidation, apoptosis, and fibrosis-related pathways [11,12]. In diabetic nephropathy, carnosic acid was also reported to improve kidney injury-related changes through activation of the Nrf2/ARE pathway and inhibition of NF-κB signaling [19]. In lipopolysaccharide-induced acute kidney injury, carnosic acid treatment was associated with improvement in renal dysfunction, histological injury, inflammatory activation, oxidative stress, and tubular apoptosis [13]. However, these models differ substantially from CA-AKI, which involves medullary hypoxia, vasoconstriction, osmotic load, and direct tubular toxicity [16–18]. Therefore, although previous renal injury models provide biological support for investigating carnosic acid, their findings should not be considered directly equivalent to the present CA-AKI model.

The biochemical, histopathological, and immunohistochemical findings in the present study were directionally consistent with a lower degree of renal injury in the carnosic acid-treated group. The significant reduction in serum urea, together with numerically lower serum creatinine levels and histopathological tubular injury scores, may be compatible with attenuation of renal injury-related changes after carnosic acid administration. Similarly, the marked increase in iNOS immunoreactivity in the CA-AKI group and its numerically lower level in the carnosic acid-treated group suggest a possible association between carnosic acid treatment and the iNOS-related inflammatory/nitrosative response.

However, this finding should be interpreted cautiously. In this study, iNOS was evaluated only by semi-quantitative immunohistochemistry. Although this method provides information on tissue-level localization and relative immunoreactivity of iNOS, it does not prove that the protective effect of carnosic acid occurs directly through iNOS suppression. In addition, additional molecular and oxidative/nitrosative stress markers, including MDA, SOD, GSH, TNF-α, IL-6, nitrotyrosine, nitrite/nitrate levels, Western blot, and qPCR, were not evaluated. Therefore, interpretations regarding antioxidant or iNOS-mediated mechanisms are based on the current findings considered together with previous literature rather than on direct measurement evidence.

The timing of treatment should also be considered when interpreting the findings of this study. In the present study, carnosic acid was administered not as a prophylactic intervention but as a therapeutic protocol initiated on the day of model induction after completion of contrast agent administration. Although current clinical strategies for CA-AKI are primarily based on risk reduction and prophylaxis, post-contrast intervention options for renal injury developing after emergency or unplanned contrast exposure remain an area of investigation. Therefore, the present design provides an experimental framework for evaluating the post-contrast therapeutic potential of carnosic acid; however, these findings should not be directly extrapolated to clinical use.

An important contribution of this study is the combined evaluation of carnosic acid at biochemical, histopathological, and immunohistochemical levels in a model of contrast-associated renal injury. This multidimensional assessment allows renal injury to be examined at different levels rather than relying on a single outcome measure. Nevertheless, the present findings should be considered hypothesis-generating. Further experimental studies incorporating different dose-timing protocols, prophylactic and therapeutic administration strategies, additional molecular markers, and long-term renal outcomes are needed to better clarify the role of carnosic acid in the context of CA-AKI.

### Limitations

This study has several limitations. First, it was conducted in an experimental animal model; therefore, the direct generalizability of the findings to clinical CA-AKI processes is limited. In addition, each group included eight animals, and no formal sample size calculation or statistical power analysis was performed before the study. Accordingly, the possibility of type II error, particularly due to the small sample size, cannot be excluded.

Second, carnosic acid was evaluated using a single-dose therapeutic protocol initiated after completion of contrast agent administration. Because a carnosic-acid-only group was not included, the independent effects of carnosic acid on healthy renal tissue could not be assessed. In addition, because corn oil, the vehicle used to dissolve carnosic acid, was not administered to the control or CA-AKI groups, a possible contribution of the vehicle effect to the findings cannot be completely excluded. Since prophylactic administration, different dose ranges, and alternative treatment timings were not investigated, no conclusion can be drawn regarding the optimal administration protocol.

Third, the assessment of renal injury was limited to short-term biochemical, histopathological, and immunohistochemical parameters. Early tubular injury, oxidative stress, inflammatory, and nitrosative stress markers such as NGAL, KIM-1, cystatin C, MDA, SOD, GSH, TNF-α, IL-6, nitrotyrosine, or nitrite/nitrate were not measured. iNOS was evaluated only by semi-quantitative immunohistochemistry, and quantitative molecular validation methods such as Western blot or qPCR was not used. Therefore, mechanistic interpretations remain limited.

Finally, histopathological and immunohistochemical evaluations were performed by a single pathologist who was blinded to the experimental groups. Intra-observer or inter-observer reliability analysis was not performed. In addition, because long-term renal functional outcomes were not assessed, no inference can be made regarding the persistence of the observed effects.

## Conclusion

In this experimental model of CA-AKI, carnosic acid administration was associated with significant improvement in selected biochemical parameters, particularly urea, sodium, and creatine kinase. Serum creatinine concentrations, histopathological tubular injury scores, and iNOS H-scores were numerically lower in the carnosic acid-treated group than in the untreated CA-AKI group; however, these pairwise differences did not reach statistical significance. Therefore, the present findings suggest a possible biochemical benefit of carnosic acid in experimental CA-AKI but do not establish a definitive histopathological or iNOS-mediated protective effect. Further studies incorporating molecular validation, different dose–timing protocols, and translational experimental designs are needed to confirm its renoprotective potential and mechanisms.

## Data Availability

The datasets used and/or analyzed during the current study are available from the corresponding author on reasonable request.
